# Ghrelin-Reactive Immunoglobulins in Conditions of Altered Appetite and Energy Balance

**DOI:** 10.3389/fendo.2017.00010

**Published:** 2017-01-27

**Authors:** Sergueï O. Fetissov, Nicolas Lucas, Romain Legrand

**Affiliations:** ^1^INSERM UMR1073, Nutrition, Gut and Brain Laboratory, Rouen, France; ^2^Institute for Research and Innovation in Biomedicine (IRIB), University of Rouen Normandy, Rouen, France

**Keywords:** ghrelin, desacyl-ghrelin, anorexia, obesity, autoantibodies

## Abstract

Part of circulating ghrelin is bound to immunoglobulins (Ig) protecting it from degradation and preserving its functional activity. This review summarizes the data on ghrelin- and desacyl-ghrelin-reactive IgG in conditions of altered appetite and energy balance. Plasma levels and affinity kinetics of such IgG were compared in patients with obesity and anorexia nervosa (AN) and in animal models of obesity including *ob/ob* mice, high-fat diet-induced obese mice, and obese Zucker rats as well as in mice after chronic food restriction and activity-based anorexia and in rats with methotrexate-induced anorexia. We show that plasmatic IgG in both obese humans and animals are characterized by increased affinity for ghrelin. In contrast, patients with AN and anorectic rodents all show lower affinity of ghrelin- and desacyl-ghrelin-reactive IgG, respectively, the changes which were not observed in non-anorectic, chronically starved mice. We also show that affinity of ghrelin-reactive IgG correlate with plasma levels of ghrelin. These data point to common mechanisms underlying modifications of affinity kinetics properties of ghrelin-reactive IgG during chronic alterations of energy balance in humans and rodents and support a functional role of such autoantibodies in ghrelin-mediated regulation of appetite.

## Introduction

Ghrelin is a 28 amino acid acylated enteroendocrine peptide produced mainly in the stomach, which has been isolated and named based on its ability to stimulate growth hormone secretion (1). One of the main properties of ghrelin, independent from growth hormone secretagogue activity, is stimulation of feeding and body weight gain (2–4). Importantly, acylation of the serine 3 residue in the ghrelin’s N-terminal by octanoic acid is necessary for binding of the growth hormone secretagogue receptor 1 (GHSR1), designated as ghrelin receptor (5) and biological activity including stimulation of food intake (6–8). However, the active, acylated form of ghrelin is unstable in the circulation and is degraded to desacyl-ghrelin (9, 10). The half-life pharmacokinetics properties of acyl-ghrelin in human plasma are about 10 min (11). The preservation of the acylated ghrelin form in the circulation appears, hence, as a key factor for maintaining its role in regulation of appetite and energy balance. Several mechanisms are involved in the regulation of ghrelin signaling during its production and receptor activation, which can be exploited for development of ghrelin-based therapy of altered appetite (12). Recently, a new regulatory factor of the ghrelin signaling has been identified, which is ghrelin-reactive immunoglobulins (Ig) (13). This review is the first attempt to summarize the data on ghrelin-reactive IgG obtained since their initial identification and the following studies in animal models of altered appetite and energy balance.

## Methods

**Figure 1:**
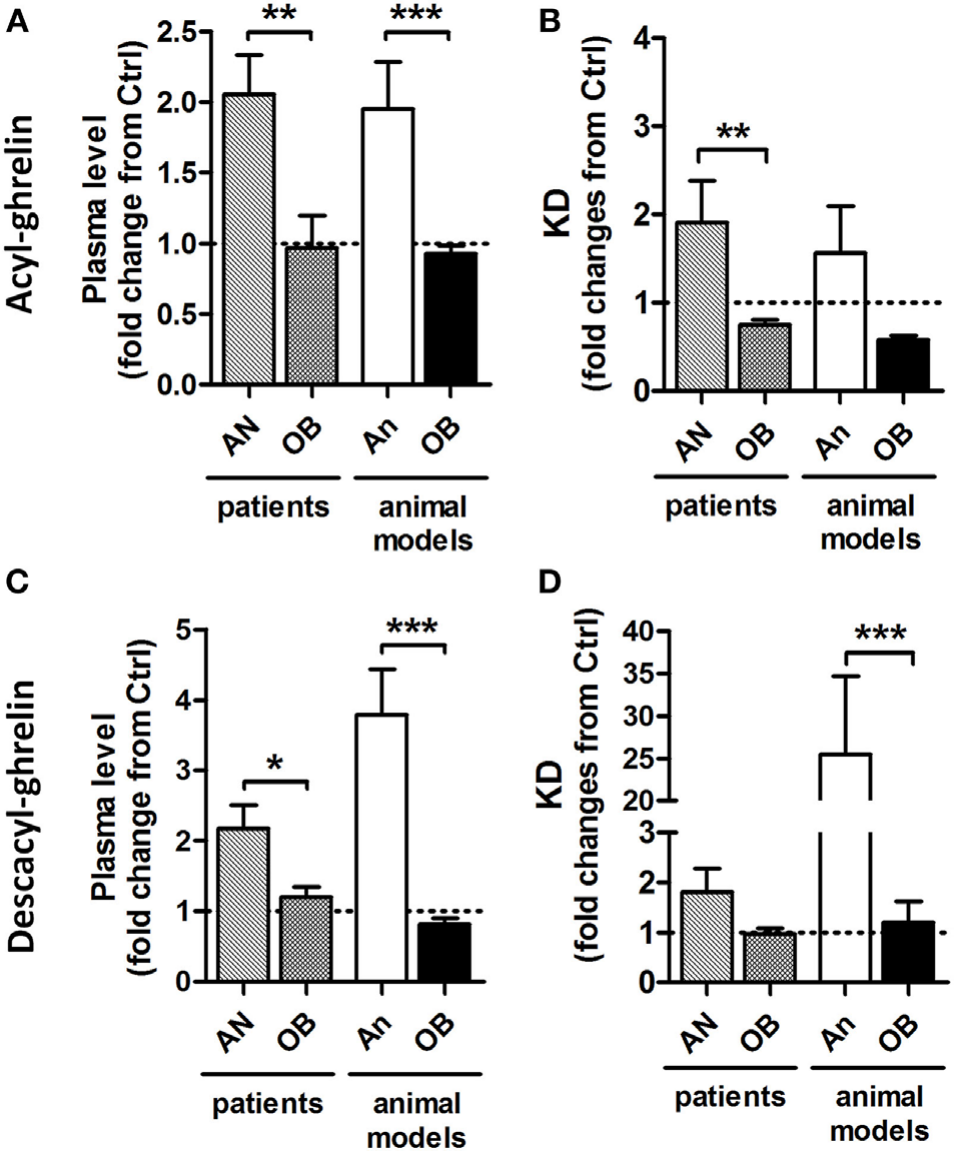
**Relative to control (Ctrl) values (1.0) changes in plasma ghrelin and desacyl ghrelin as well as affinity of their reactive IgG in patients and animal models of obesity and anorexia**. Changes in plasma levels of acyl-ghrelin **(A)** and desacyl-ghrelin **(C)**. Changes in affinity (dissociation equilibrium constant, KD) of plasmatic IgG for acyl-ghrelin **(B)** and desacyl ghrelin **(D)**. AN, anorexia nervosa patients; An, animal models of anorexia; OB, obese patients or animals. Mann–Whitney tests ***p < 0.001, **p < 0.01, and *p < 0.05.

The review focuses mainly on the data from five papers, all reporting affinity kinetics of ghrelin- and desacyl-ghrelin-reactive IgG. They include a study of patients with hyperphagic obesity and anorexia nervosa (AN) and of *ob/ob* mice by Takagi et al. (13); two studies by François et al. in high-fat diet (HFD)-induced obese mice (14) and in rats with methotrexate (MTX)-induced anorexia (15), a study by Lucas et al. in obese Zucker rats (16) as well as a Legrand et al. study in mice after chronic food-restriction and activity-based anorexia (17). In all mentioned studies, the same protocols of IgG plasma extraction and affinity kinetic assay by the surface plasmon resonance were used as was previously described in details (18). All measurements of ghrelin and desacyl-ghrelin peptides in above mentioned studies were performed using corresponding kits from Mitsubishi Chemical Med Corp. (Tokyo, Japan), selectively detecting acyl- and desacyl-ghrelin with resulting plasma ratios of 1:3 to 1:6 in humans. In the text, we use the terms of ghrelin and acyl-ghrelin as synonyms. For the statistical analysis of ghrelin and ghrelin-reactive IgG properties between different animal models and patients, they are shown as means of folds of changes vs. corresponding controls. In this analysis, we combined two animal models of anorexia including ABA mice and MTX rats and three animal models of obesity including *ob/ob* mice, HFD-induced obese mice, and obese Zucker rats. Group differences shown in Tables 1–4 and Figure 1 were analyzed using statistical tests indicated in the legends. We also used the Pearson’s test to analyze correlations between plasma levels of ghrelin or desacyl-ghrelin and plasma levels and affinity (KD values) of corresponding IgG.

**Table 1:** **Plasma concentrations of acyl-ghrelin and desacyl-ghrelin in humans and rodents**.

	Acyl-ghrelin (pM)		Desacyl-ghrelin (pM)		Acyl/desacyl-ghrelin ratio	
**Mean ± SEM**						
OB	24.88 ± 5.81	ns	137.7 ± 16.07	ns	1:6	↘^$^
AN	52.67 ± 7.14	↗^$$$^	249.1 ± 38.32	↗^$$$^	1:4	ns
Ctrl	25.65 ± 3.58		114.7 ± 29.24		1:3	
*ob/ob*	10.36 ± 0.96	↘^#^	75.17 ± 10.31	↘*	1:7	ns
Ctrl	13.02 ± 0.90		120.4 ± 17.32		1:8	
HFD	10.21 ± 0.95	ns	117.3 ± 9.75	↘**	1:10	ns
Ctrl	11.92 ± 1.45		196.5 ± 27.95		1:13	
Zucker	17.18 ± 1.59	ns	444.8 ± 33.86	↗*	1:25	ns
Ctrl	16.20 ± 4.38		242.0 ± 55.80		1:16	
ABA	21.58 ± 6.86	ns	1331.0 ± 87.52	↗^$$^	1:63	ns
FTR	17.82 ± 5.02	ns	1221.0 ± 120.7	↗^$^	1:71	↘^$^
Ctrl	9.14 ± 2.50		195.8 ± 22.50		1:17	
MTX	12.68 ± 2.21	↗**	698.8 ± 95.8	↗*	1:46	ns
Ctrl	7.44 ± 1.08		352.4 ± 27.79		1:46	

Human patients: OB, obese; AN, anorexia nervosa. Rodent models of obesity: ob/ob mice; HFD, high fat diet-induced obese mice and Zucker rats. Rodent models of anorexia and starvation: ABA, activity-based anorexia in mice; FTR, feeding-time restriction in mice; and MTX, methotrexate-induced anorexia in rats. Ctrl, healthy human or rodent controls in corresponding row. Student’s t-test or Mann–Whitney test, *p < 0.05; **p < 0.01; ^#^p < 0.10. ANOVA with Tukey’s posttest or Kruskal–Wallis with Dunn’s posttest ^$^p < 0.05, ^$$^p < 0.01, ^$$$^p < 0.001.

**Table 2:** **Plasma levels of IgG reactive with acyl-ghrelin and desacyl-ghrelin in humans and rodents**.

	Acyl-ghrelin-reactive IgG				Desacyl-ghrelin-reactive IgG				Acyl-ghrelin-reactive IgG		Desacyl-ghrelin-reactive IgG	
	Free		Total		Free		Total		Free/total ratio		Free/total ratio	
**Mean ± SEM**												
OB	0.291 ± 0.050	ns	0.617 ± 0.054	↘^$^	0.563 ± 0.049	ns	1.250 ± 0.057	ns	1:2.1	↗^$^	1:2.3	ns
AN	0.288 ± 0.020	ns	0.753 ± 0.049	ns	0.643 ± 0.054	ns	1.364 ± 0.080	ns	1:2.5	ns	1:2.1	ns
Ctrl	0.264 ± 0.020		0.918 ± 0.103		0.584 ± 0.094		1.384 ± 0.101		1:3.2		1:2.4	
*ob/ob*	0.133 ± 0.025	↘*	0.098 ± 0.013	↘^#^	n.a.	n.a.	1:0.6	ns	n.a.			
Ctrl	0.207 ± 0.024		0.162 ± 0.028		n.a.	n.a.			1:0.8		n.a.	
HFD	0.504 ± 0.050	ns	0.450 ± 0.033	↘**	n.a.	n.a.	1:0.8	ns	n.a.			
Ctrl	0.589 ± 0.067		0.624 ± 0.059		n.a.	n.a.			1:0.9		n.a.	
Zucker	0.268 ± 0.062	↘*	0.235 ± 0.073	↘*	0.244 ± 0.048	↘^#^	0.241 ± 0.066	↘*	1:0.6	↗*	1:0.8	↗*
Ctrl	0.715 ± 0.206		1.061 ± 0.259		0.627 ± 0.165		0.975 ± 0.225		1:1.5		1:1.5	
ABA	0.258 ± 0.084	ns	0.340 ± 0.127	ns	0.226 ± 0.091	ns	0.366 ± 0.127	ns	1:1.5	↗^£^	1:1.4	ns
FTR	0.187 ± 0.045		0.459 ± 0.109		0.242 ± 0.062		0.501 ± 0.127		1:2.4		1:2.0	
Ctrl	0.172 ± 0.052		0.315 ± 0.043		0.238 ± 0.069		0.464 ± 0.080		1:1.9		1:2.0	
MTX	0.063 ± 0.016	↘*	0.041 ± 0.007	↘***	0.070 ± 0.015	↘*	0.031 ± 0.006	↘***	1:0.6	↗***	1:0.4	↗***
Ctrl	0.188 ± 0.043		0.319 ± 0.044		0.223 ± 0.048		0.271 ± 0.036		1:1.7		1:1.2	

Human patients: OB, obese; AN, anorexia nervosa. Rodent models of obesity: ob/ob mice; HFD, high fat diet-induced obese mice and Zucker rats. Rodent models of anorexia and starvation: ABA, activity-based anorexia in mice; FTR, feeding-time restriction in mice; and MTX, methotrexate-induced anorexia in rats. Ctrl, healthy human or rodent controls in corresponding row. Student’s t-test or Mann–Whitney test, *p < 0.05; **p < 0.01; ***p < 0.001; ^#^p < 0.10. ANOVA with Tukey’s posttest or Kruskal–Wallis test with Dunns’ posttest, ^$^p < 0.05; ABA vs. FTR ^£^p < 0.05.

**Table 3:** **Affinity kinetics of acyl-ghrelin-reactive IgG in humans and rodents**.

	Acyl-ghrelin-reactive IgG					
	Ka (1/Ms)		Kd (1/s)		KD (M)	
**Mean ± SEM**						
AN	9.36E+04 ± 2.23E+03	ns	3.25E−03 ± 8.04E−04	↗^$^	4.86E−07 ± 1.20E−07	↗^§§^
OB	2.23E+04 ± 1.16E+04	↗^$^	1.86E−03 ± 3.04E−04	ns	1.91E−07 ± 1.48E−08	ns
Ctrl	6.84E+03 ± 1.19E+03		1.49E−03 ± 1.14E−04		2.54E−07 ± 2.81E±08	
ob/ob	1.01E+05 ± 9.46E+03	ns	1.81E−03 ± 7.81E−05	↘**	2.03E−08 ± 2.88E−09	↘*
Ctrl	8.89E+04 ± 9.08E+03		2.46E−03 ± 1.48E−04		3.02E−08 ± 3.57E−09	
HFD	1.68E+04 ± 4.87E+03	ns	1.58E−03 ± 3.22E−04	ns	1.17E−07 ± 1.18E−08	↘**
Ctrl	1.04E+04 ± 2.06E+03		2.02E−03 ± 2.82E−04		2.55E−07 ± 4.51E−08	
Zucker	1.01E+04 ± 1.54E+03	ns	1.22E−03 ± 1.61E−04	↘*	1.36E−07 ± 2.65E−08	↘*
Ctrl	7.93E+03 ± 6.53E+02		1.70E−03 ± 9.79E−05		2.24E−07 ± 2.50E−08	
ABA	4.72E+04 ± 8.12E+03	↗*^,£^	5.11E−04 ± 1.07E−04	ns	1.24E−08 ± 2.83E−09	ns
FTR	2.03E+04 ± 6.62E+03	ns	2.51E−04 ± 1.32E−04	ns	1.38E−08 ± 5.61E−09	ns
Ctrl	1.48E+04 ± 5.58E+03		2.45E−03 ± 1.15E−04		1.52E−08 ± 6.15E−09	
MTX	2.00E+03 ± 6.30E+02	ns	1.29E−03 ± 2.28E−04	↗*	1.18E−07 ± 4.92E−07	ns
Ctrl	1.79E+03 ± 5.06E+02		5.97E−04 ± 1.92E−04		5.73E−07 ± 2.49E−08	

Human patients: OB, obese; AN, anorexia nervosa. Rodent models of obesity: ob/ob mice; HFD, high fat diet-induced obese mice and Zucker rats. Rodent models of anorexia and starvation: ABA, activity-based anorexia in mice; FTR, feeding-time restriction in mice; and MTX, methotrexate-induced anorexia in rats. Ctrl, healthy human or rodent controls in corresponding row. ANOVA with Tukey’s posttest or Kruskal–Wallis test with Dunn’s posttest, ^$^p < 0.05; AN vs. OB ^§§^p < 0.01. Student’s t-test or Mann–Whitney test vs. Ctrl *p < 0.05; **p < 0.01, vs. RFA ^£^p < 0.05.

**Table 4:** **Affinity kinetics of desacyl-ghrelin-reactive IgG in humans and rodents**.

	Desacyl-ghrelin-reactive IgG					
	Ka (1/Ms)		Kd (1/s)		KD (M)	
**Mean ± SEM**						
AN	1.04E+04 ± 1.02E+03	ns	2.26E−03 ± 3.86E−04	ns	8.61E−07 ± 7.17E−08	ns
OB	2.21E+04 ± 9.80E+03	ns	1.74E−03 ± 4.16E−04	ns	1.47E−07 ± 1.69E−08	ns
Ctrl	1.21E+04 ± 1.28E+03		1.65E−03 ± 1.50E−04		1.51E−07 ± 1.56E−08	
ob/ob	3.73E+03 ± 3.61E+03	↘^#^	7.23E−03 ± 8.10E−07	ns	9.59E−06 ± 3.76E−06	ns
Ctrl	1.89E+04 ± 9.33E+03		2.68E−05 ± 1.55E−05		4.13E−06 ± 3.38E−06	
HFD	2.72E+03 ± 1.74E+03	ns	1.34E−04 ± 1.30E−04	↘*	3.79E−08 ± 1.47E−08	↘^#^
Ctrl	1.09E+04 ± 4.71E+03		8.39E−04 ± 3.03E−04		1.38E−07 ± 4.72E−08	
Zucker	2.40E+04 ± 3.55E+03	ns	1.34E−03 ± 3.51E−04	↘*	6.78E−08 ± 2.30E−08	ns
Ctrl	2.85E+04 ± 4.24E+03		2.26E−03 ± 3.05E−04		9.88E−08 ± 2.21E−08	
ABA	1.94E+04 ± 8.06E+03	ns	1.12E−03 ± 2.09E−04	↗^$$^	9.20E−08 ± 2.34E−08	↗^$^
FTR	8.32E+03 ± 2.89E+03	ns	3.24E−04 ± 2.03E−04	ns	1.03E−07 ± 5.97E−08	ns
Ctrl	1.74E+04 ± 9.64E+03		2.07E−06 ± 1.20E−06		1.57E−08 ± 1.06E−09	
MTX	2.56E+03 ± 1.55E+03	ns	2.01E−04 ± 1.33E−04	ns	6.97E−08 ± 1.73E−08	↗*
Ctrl	1.71E+02 ± 1.33E+02		1.05E−06 ± 2.16E−07		2.07E−08 ± 5.46E−09	

Human patients: OB, obese; AN, anorexia nervosa. Rodent models of obesity: ob/ob mice; HFD, high fat diet-induced obese mice and Zucker rats. Rodent models of anorexia and starvation: ABA, activity-based anorexia in mice; FTR, feeding-time restriction in mice; and MTX, methotrexate-induced anorexia in rats. Ctrl, healthy human or rodent controls in corresponding row. ANOVA with Tukey’s posttest or Kruskal–Wallis test with Dunn’s posttest, ^$^p < 0.05; ^$$^p < 0.01. Student’s t-test or Mann–Whitney test, vs. Ctrl *p < 0.05; ^#^p < 0.10.

## Detection of Ghrelin-Reactive IgG

Ghrelin-reactive IgG were first shown to be naturally present in plasma of healthy humans and rodents in 2008 by Fetissov et al. (19). Further studies confirmed and extended this initial finding by including female patients with restrictive AN (20) and with hyperphagic obesity (13). Moreover, a study using plasma samples of a large number of healthy male (*n* = 562) and female (*n* = 636) adolescents also revealed an ubiquitous presence of ghrelin-reactive autoantibodies of both IgG and IgM classes, which mean plasma levels were slightly elevated in girls vs. boys (21). In rodents, ghrelin-reactive IgG were detected in both rats (15, 19) and mice (13, 14, 17). Since the rodents studies were performed only in males, presence of possible sex differences of ghrelin-reactive IgG was not explored. However, it is likely that similarly to humans they may also be increased in female rodents who naturally display elevated plasma levels of total IgG and IgM as well as increased levels of autoantibodies reactive with other neuropeptides, e.g., α-melanocyte-stimulating hormone (α-MSH) (22).

Ghrelin-reactive IgG were also measured in the hypothalamic and liver tissue of C57Bl6 mice, showing their levels at the limit of detection (13). However, if levels of ghrelin-reactive IgG in these tissues of lean mice were stratified according to their exposure to a restraint stress in an EchoMRI instrument, which they had undergone prior to tissue sample for the analysis of their body composition, an increase is observed in both tissues and a decrease in plasma of stressed animals. It suggests that ghrelin-reactive IgG may help transportation of ghrelin mobilized during stress from plasma to its tissue targets.

Desacyl-ghrelin-reactive IgG are also present in humans (13, 20) and rodents (15, 17). Although some part of desacyl-ghrelin-reactive IgG may bind to acyl-ghrelin, they should also have distinct paratopes, because levels of desacyl-ghrelin IgG are often higher than of acyl-ghrelin IgG in human plasma (13, 20) and also because they have different affinity kinetics properties (13). The exact epitopes responsible for plasma IgG binding to ghrelin and desacyl-ghrelin remain to be been studied.

Both “free” and “total” ghrelin- and desacyl-ghrelin-reactive IgG are detected in plasma of humans and rodents. While “free” IgG are measured in physiological buffer, the “total” levels are measured in a high salt buffer, which dissociate immune complexes typically resulting in increased “total” vs. “free” levels of IgG. Accordingly, increased levels of free/total ratios may reflect an increase in IgG available to bind ghrelin or desacyl-ghrelin (Table 2).

## Origin and Regulation of Ghrelin-Reactive IgG

Ghrelin-reactive IgG are natural autoantibodies and, therefore, are produced by germ-like B-cells as a part of native immunity (23). As was postulated by Avrameas, natural autoantibodies may participate in a complex regulatory network, not necessarily directly related to the immune function but contributing to the homeostatic control (24). The production and affinity maturation of IgG are influenced by a variety of antigens and non-specific immune-stimulatory or inhibitory factors such as cytokines and steroid hormones. Moreover, increased plasma levels of ghrelin-reactive IgG in rats can be induced acutely by gastric electrical stimulation (25), suggesting that they can be stored and released upon a physiological stimulus. It is of interest that gastric electrical stimulation activated c-fos production in numerous lymphoid cells of the gastric mucosa surrounding the enteroendocrine ghrelin-synthetizing cells (26). Strong inhibition of ghrelin-reactive IgG levels in plasma is observed in rats after treatment with MTX, an immunosuppressive agent, which induces intestinal mucositis (15).

A particular role in production of IgG and other classes of Ig can be played by antigens derived from gut microbiota as a constitutive part of the gastro-intestinal tract in all animals (27). The gut bacterial antigens may also be involved in regulation of production and properties of ghrelin-reactive IgG. In fact, germ-free rats showed elevated plasma levels of ghrelin-reactive IgG (19), supporting a role of some intestinal antigens in tolerization toward ghrelin (28). The molecular basis linking microbial antigens with peptides may relate to the concept of molecular mimicry by cross-reactive autoantibody production (29). Indeed, sequence homology between ghrelin and several proteins from commensal gut microorganisms was shown, e.g., of seven consecutive amino acids present in a protein from *Enterococcus faecalis* (19). A role of molecular mimicry was recently validated as a mechanism underlying production of α-MSH-reactive IgG induced by caseinolytic peptidase B (ClpB) homolog protein from *Escherichia coli* (30). ClpB contains a discontinuous six amino acid homology with α-MSH; immunization of mice with ClpB generates α-MSH-cross reactive IgG (30). Plasma presence of the IgA class of ghrelin-reactive IgG also points to their origin triggered by luminal antigens (19).

Beside the stimulation of antigens from commensal gut microbiota, pathogenic and environmental microorganisms may possibly influence production of ghrelin-reactive IgG. In fact, plasma levels of ghrelin-reactive IgG in healthy adolescents correlate strongly with both IgG and IgM directed against influenza A virus (21). Although the initial *in silico* search for five consecutive amino acids sequence homology did not reveal matches between ghrelin and the Influenza viruses (19), a discontinuous eight amino acid homology for ghrelin is present in the PB1-F2 influenza A protein.

Another example of ghrelin-reactive IgG comes from studies of children with idiopathic short stature by the Lewinski group. They showed that prevalence of high level IgG reactive with several peptide hormones, including ghrelin, orexin A, leptin, and α-MSH combined, was associated with an increased incidence of *Helicobacter pylori* and/or *Candida albicans* (31). A follow-up study showed that mean plasma levels of ghrelin-reactive IgG were also elevated in children with short stature, but they were not significantly different in patients with growth hormone deficiency (32). Since *H. pylori* is only residing in the stomach, which is the main source of ghrelin, it is of interest to explore possible role of *H. pylori* in production of ghrelin-reactive IgG. In fact, cure of *H. pylori* in humans was accompanied by increased plasma levels of ghrelin (33).

## Functional Role of Ghrelin-Reactive IgG

An established physiological role of IgG is to protect against infection including their ability to directly neutralize antigens and to trigger cell lysis *via* activation of the complement. While presence of IgG reactive with some peptide hormones have been detected in plasma of healthy humans (34), their possible functional role remained unknown, as previously reviewed (35). Ghrelin-reactive IgG were the first IgG revealing that they have a functional role in the peptide signaling by protecting ghrelin from degradation in plasma (13). In fact, as was shown by an *in vitro* assay, depletion of plasma from IgG resulted in an almost complete loss of acyl-ghrelin, while their reintroduction allowed its full recovery (13). Thus, the main functional role of ghrelin-reactive IgG appears to protect ghrelin from degradation.

Such protection did not reduce the orexigenic activity of ghrelin, because administrations of ghrelin together with plasmatic IgG, stimulated food intake and even enhanced it when IgG were derived from plasma of obese humans or mice (13). Such enhancing effect was explained by a slight, about three times, increase of IgG affinity for ghrelin and the evidence that such IgG are able to transport more active ghrelin in obese patients. Moreover, the KD values of ghrelin-reactive IgG remained in the micromolar range, which would preclude their competition with the nanomolar affinity of ghrelin receptor binding (36).

**Figure 2:**
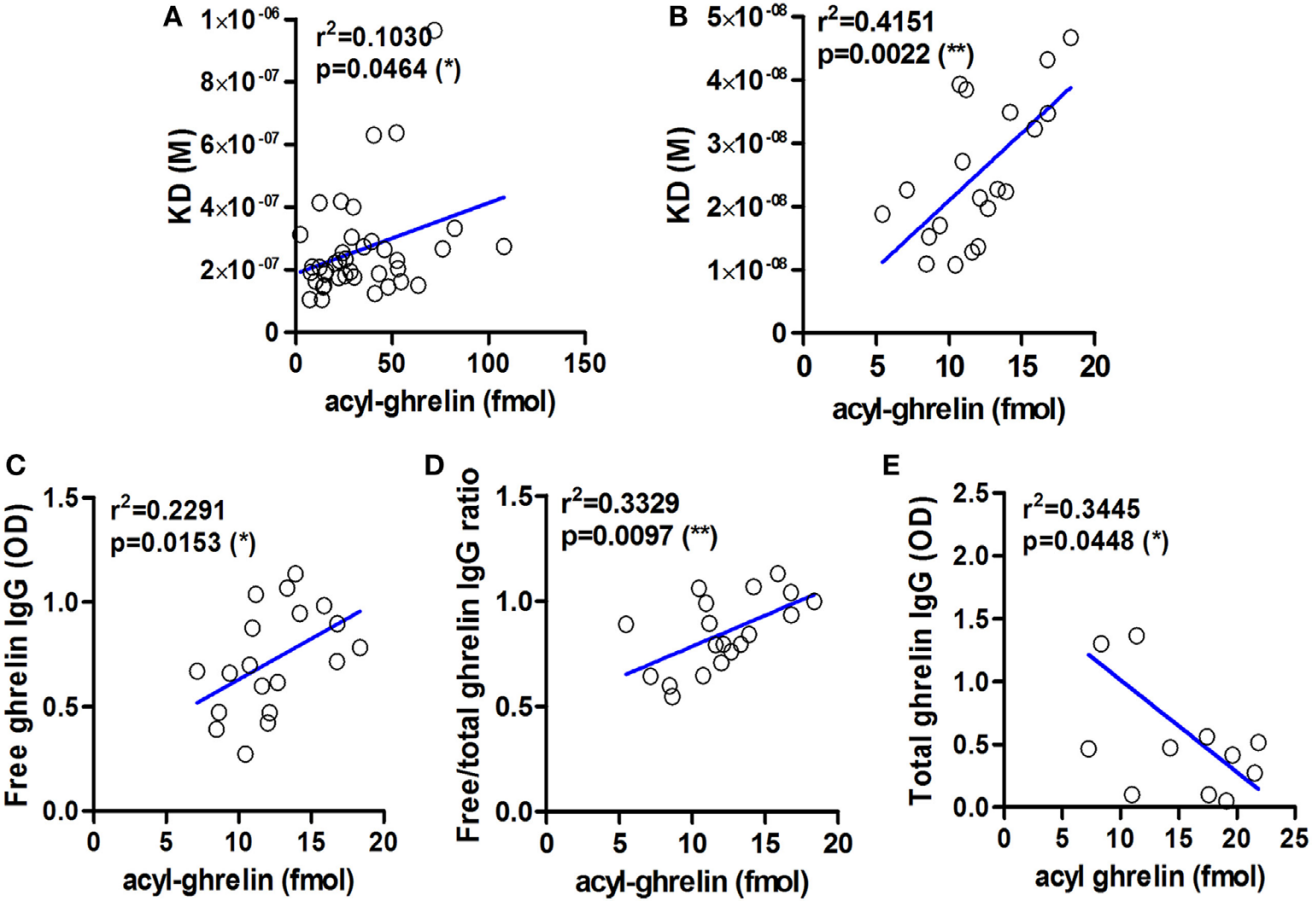
**Examples of correlations between affinity and levels of ghrelin-reactive IgG and plasma concentrations of acyl-ghrelin**. Correlations between the values of dissociation equilibrium constants (KD) of plasmatic IgG reactive with ghrelin and acyl ghrelin in obese and anorectic patients and controls **(A)** and in *ob/ob* and lean mice **(B)**. Correlations between plasma levels of ghrelin-reactive free IgG **(C)** as well as ratios of their free/total levels **(D)** in ob/ob and lean mice. Correlations between plasma levels of ghrelin-reactive total IgG in Zucker rats **(E)**. *R*-squared and *p*-values for Pearson’s correlation tests are shown, **p < 0.01 and *p < 0.05.

We show here that KD values of IgG for ghrelin correlate positively with plasma ghrelin concentrations in obese and AN patients as well as in *ob/ob* mice (Figures 2A,B). These correlations demonstrate that a decrease in IgG affinity for ghrelin (increase in KD) corresponds to higher ghrelin levels and suggest that properties of ghrelin-reactive IgG may at least partly underlie individual differences in plasma ghrelin levels. An inverse relation is unlikely, because increased antigen concentration should stimulate the affinity maturation of IgG. We also find that plasma levels of ghrelin-reactive free IgG as well as ratios of free/total IgG levels correlate positively with plasma ghrelin (Figures 2C,D). In contrast, ghrelin-reactive total IgG correlate negatively with plasma ghrelin (Figure 2E). Taken together, these correlations support a carrier role of plasmatic IgG for ghrelin.

Considering increased functional effects of ghrelin in immune complex with IgG vs. ghrelin alone, the accompanying changes in plasma ghrelin levels during altered energy metabolism can be a “mirror” reflection of the ghrelin’s functional activity. Accordingly, increased plasma levels of ghrelin, e.g., in AN patients and in animal models of anorexia (Figure 1), may signify ghrelin’s inability to form stable immune complexes with IgG and, hence, may result in a functional deficiency of the ghrelin signaling or “ghrelin resistance.” Such explanation may appear at first glance as paradoxical, but it gains further support from the corroborating data revealing an enhancing role of IgG in signaling by other peptide hormones such as α-MSH (37, 38).

Thus, the changes in IgG affinity kinetics resulting in a slight increase of affinity can be considered as a gain of function in ghrelin signaling. Taking in account important functional consequences of affinity changes in ghrelin-reactive IgG, it is clear that a simple measurement of their plasma levels is not sufficient and should be combined with the affinity kinetics analysis. Nevertheless, affinity changes should still be taken with caution as a putative biomarker, as long as we do not know if they involve or not any epitope changes that may prevent the availability of the ghrelin N-terminal, necessary for the GHSR1 binding. We do not yet know if presence of IgG in immune complex with ghrelin may play an allosteric role in ghrelin receptor activation, a phenomenon suggested for IgG reactive with α-MSH (37, 38).

To summarize these data, presence of IgG at normal levels and with physiological micromolar affinity for ghrelin in humans appears as a homeostatic factor in regulation of ghrelin signaling. Accordingly, any changes in these factors should lead to alteration of the ghrelin signaling with resulting effects on appetite and body weight regulation. Therefore, in order to facilitate the data interpretation toward an enhanced or diminished ghrelin signaling, the following algorithm can be proposed.

For the enhanced ghrelin signaling one of several changes can be observed:

– Increased plasma levels of ghrelin-reactive both free and total IgG;
– Increased micromolar affinity of IgG for ghrelin;
– Changes in affinity kinetics of IgG for ghrelin toward its increased binding including increased association rate (small Ka) and/or decreased dissociation rate (small Kd).

By the opposite, for the diminished ghrelin signaling, the following changes can be present:

– Decreased plasma levels of ghrelin-reactive both free and total IgG;
– Decreased micromolar affinity of IgG for ghrelin;
– Changes in affinity kinetics of IgG for ghrelin toward its decreased binding including decreased association rate (small Ka) and/or increased dissociation rate (small Kd).

While the functional role of ghrelin-reactive IgG with regard to ghrelin’s orexigenic effect can be interpreted as facilitating, the corresponding role of IgG reactive with desacyl-ghrelin is uncertain. As discussed above, IgG may bind to the common central and the C-terminal parts of ghrelin and desacyl-ghrelin and, hence, may transport both types of peptides. In fact, absorption studies of ghrelin- and desacyl-ghrelin-reactive IgG confirmed that they can bind both peptides (20).

Although some studies showed potential anorexigenic effects of desacyl-ghrelin (39, 40), its functional role in the regulation of appetite remains uncertain including direct functional consequences of desacyl-ghrelin transport by IgG toward an unknown putative receptor (41). One alternative possibility is that desacyl-ghrelin may compete with acyl-ghrelin for protective IgG and, thereby, desacyl-ghrelin may have an anorexigenic effect by diminishing the ghrelin signaling *via* reduced formation of ghrelin/IgG immune complexes. Such possibility is of functional importance because starvation preferentially upregulates desacyl-ghrelin, e.g., its ratios to acyl-ghrelin can be increased from 1:20 in *ad libitum* fed mice to more than 1:70 in starved mice (Table 1) (17). Functional significance of the affinity kinetics changes in desacyl-ghrelin-reactive IgG is also difficult to interpret owning the same problem of the uncertain role of the desacyl-ghrelin peptide. Whether such changes are different from those obtained for acyl-ghrelin, it suggests that they may concern antibodies primarily targeting the desacylated N-terminal of ghrelin. It is, nevertheless, possible that decreased affinity of IgG for desacyl-ghrelin may be favorable for desacyl-ghrelin competition with ghrelin for ghrelin-protective IgG and, hence, may result in reduced orexigenic activity of ghrelin. Furthermore, we have seen that KD values of desacyl-ghrelin IgG correlate positively with plasma levels of desacyl-ghrelin in animals with anorexia. Significant correlations were also found between plasma levels of desacyl-ghrelin reactive IgG and desacyl-ghrelin in patients with obesity and AN. Such correlations suggest that, similar to acyl-ghrelin, desacyl-ghrelin plasma concentrations can also be regulated by desacyl-ghrelin-reactive IgG.

## Ghrelin-Reactive-IgG in Obesity

Obesity is typically accompanied by increased food intake, suggesting a possible role of ghrelin in the mechanism of hyperphagia (42). Indeed, obese subjects are more sensitive than lean subjects to increase their food intake after ghrelin administration (43). A particular situation may exist in non-hyperphagic HFD-induced obesity in mice that are less sensitive to the ghrelin’s orexigenic effect (44). In obese subjects, basal plasma levels of ghrelin measured as total ghrelin (acyl-ghrelin + desacyl-ghrelin) were found low (45, 46) or normal when measured selectively for acyl-ghrelin (13, 47, 48) (Table 1). Thus, increased food intake in common obesity cannot be related to increased ghrelin concentrations. There are, nevertheless, some exceptions showing elevated plasma ghrelin in hyperphagic obese patients with the Prader–Willi syndrome (PWS) (49) and hyperphagic obese Zucker rats (50, 51). However, our study showed that obese Zucker rats display elevated desacyl-ghrelin, while acyl-ghrelin remains normal (16) (Table 1).

IgG in obese patients showed their ability to better protect ghrelin from degradation by an *in vitro* test and to increase its orexigenic activity after intraperitoneal administrations in rats (13). Such ghrelin’s enhancing properties of IgG in obesity can be explained by its increased affinity, as discussed above. Similarly, increased affinity of IgG for ghrelin was found in genetic animal models of obesity including both *ob/ob* mice (13) and Zucker rats (16) (Table 3). Obesity in both rodent’s models is due to the deficient leptin signaling and is characterized by hyperphagia. In mice that developed obesity after 2 months of HFD, IgG was also characterized by increased affinity for ghrelin (Table 3); however, these mice are not hyperphagic. In contrast to other animal models of obesity and to obese patients, HFD-obese mice also showed an increase in affinity of desacyl-ghrelin IgG (Table 4), but the significance of such change is not clear. The affinity kinetic properties of the association and dissociation rates leading to increased affinity were all different between humans and animal models of obesity including increased small *Ka* in obese patients, a decrease of small *Kd* in *ob/ob* mice and Zucker rats, and no significant changes in HFD obese mice (Table 3).

Although plasma levels of ghrelin-reactive IgG in obese patients and in all animal models reviewed here were slightly lower than in their non-obese controls (Table 2), the free/total ratios of ghrelin-reactive IgG were increased in patients and in Zucker rats (Table 2). Interestingly, a recent conference report revealed elevated plasma levels of ghrelin-reactive IgG in children with PWS (52). If confirmed, it will be the only so far known pathological conditions characterized by increased production of ghrelin-reactive IgG.

Taken together, these data suggest that obesity development leads to increased carrying properties of IgG for ghrelin in both obese humans and in animal models of obesity *via* increased affinity (Figure 1). Such changes in ghrelin-reactive IgG properties may represent a mechanistic factor underlying enhanced ghrelin signaling leading to hyperphagia and increased adiposity.

## Ghrelin-Reactive-IgG in Anorexia

Anorexia can be a primary problem in patient with eating disorders such as in restrictive AN, or it can be symptomatic during a chronic disease worsening its outcome. Ghrelin production and its plasma concentration are typically elevated during chronic starvation, accompanied or not by anorexia in both humans and experimental animals (13, 17, 53–56). Such elevation of plasma ghrelin suggests its homeostatic role in the long-term regulation of energy balance aimed at increased food intake, once food will be available (42). This homeostatic control is obviously not working in anorectic humans and rodents, who display functional “ghrelin resistance.” A role of plasmatic IgG in mechanisms of “ghrelin resistance” during anorexia can be suggested based on changes in their properties in an opposite way to obesity. In fact, patients with AN display lower affinity than in controls (Table 3), and many AN patients display also low plasma levels of ghrelin-reactive IgG (20). In the ABA and MTX rodent models of anorexia, we did not observe low affinity of IgG for ghrelin but it was present for desacyl-ghrelin (Tables 3 and 4). Such decrease of affinity was detected only in ABA mice but not in FTR mice that were starved but did not develop spontaneous anorexia, suggesting a possible contribution of desacyl-ghrelin-reactive IgG in the anorectic phenotype. MTX-treated rats, which develop severe anorexia, were also characterized by strong inhibition of ghrelin-reactive IgG levels (15). As discussed above, lower levels and affinity of IgG for both ghrelin and desacyl-ghrelin may decrease ghrelin’s orexigenic effects. Such possibility was not, however, experimentally validated.

Whether IgG may improve ghrelin’s orexigenic effects was tested in the ABA model in mice, but the orexigenic effects of treatments were limited to the 3 h of daily feeding time, which was not sufficient to fully evaluate their enhancing roles (17). In any case, only IgG from obese mice were efficient to significantly improve ghrelin’s orexigenic effect.

These data suggest that factors which specifically or non-specifically decrease plasma levels and affinity of ghrelin-reactive IgG may diminish ghrelin’s orexigenic effects and, hence, will contribute to the mechanisms of anorexia *via* functional “ghrelin resistance.”

## Conclusion

**Figure 3:**
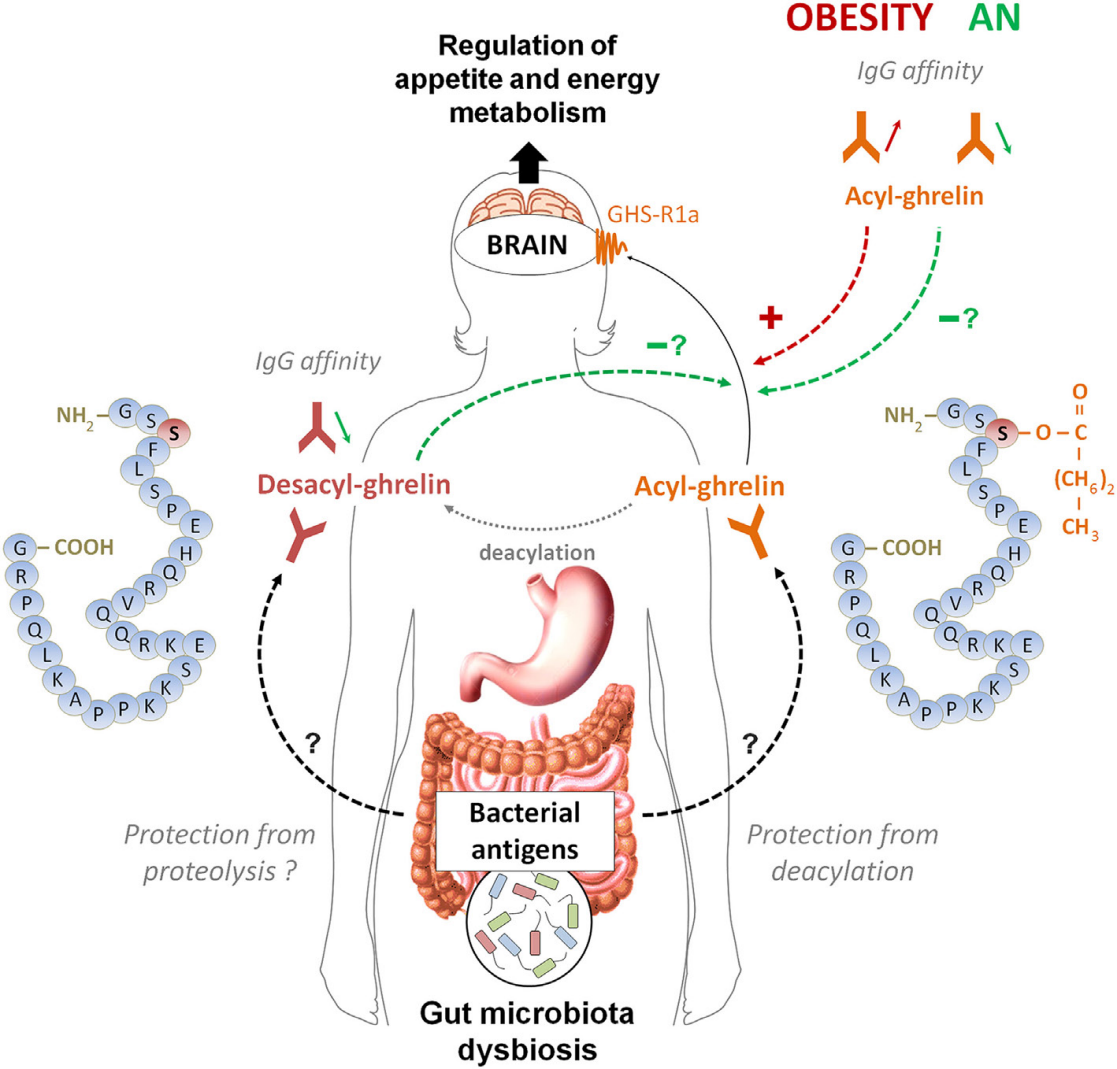
**A schematic model summarizing the postulated role of ghrelin-reactive IgG in ghrelin signaling in appetite control during normal and pathological conditions**. According this model, acyl-ghrelin is protected by IgG from deacylation, therefore enhancing its orexigenic signaling to the brain. Changes in affinity of IgG for ghrelin in obese patients (increase) and in orexia nervosa patients (decrease) may enhance and diminish, respectively, ghrelin’s orexigenic effects. Desacyl-ghrelin may lower orexigenic effect *via* competing with ghrelin for ghrelin-reactive IgG resulting in increased ghrelin degradation. Moreover, decreased affinity of IgG for desacyl ghrelin may favor its dissociation from immune complexes and completion with ghrelin. The origin of changes in affinity of ghrelin- and desacyl ghrelin-reactive IgG is currently unknown but may potentially depend on antigenic stimulation from dysbiotic gut microbiota associated with long-term nutritional modifications in anorexia and obesity.

The main conclusions that can be drawn after reviewing these data are the existence of a functional link between ghrelin-reactive IgG and plasma ghrelin levels as well as a physiological role of ghrelin-reactive IgG in modulating ghrelin’s biological activities including conditions of altered energy balance. A summary Figure 3 schematically illustrates the postulated link between ghrelin-reactive IgG and regulation of appetite whereas changes in IgG properties during obesity and anorexia may enhance or diminish ghrelin signaling, while desacyl-ghrelin may compete with ghrelin for IgG which protect ghrelin from deacylation.

Data analysis from patients and several animal models of altered energy balance shows that both obese humans and animals display lower levels of ghrelin-reactive IgG characterized by increased affinity. In contrast, patients with AN and anorectic rodents all show lower affinity of ghrelin- and desacyl-ghrelin-reactive IgG, respectively, the changes which were not observed in non-anorectic chronically starved mice. Such changes in properties of both ghrelin- and desacyl-ghrelin-reactive IgG in patients with obesity and anorexia suggest that they are not a direct consequence of the positive or negative energy balance *per se* but may be a result of some typical changes associated with altered regulation of energy metabolism impacting on the autoantibody production. Although the mechanisms of such common changes are currently unknown, they may potentially involve modifications of gut microbiota leading to altered production of ghrelin-like antigenic molecules. Indeed, typical, but not identical response of gut microbiota to starvation or an obesogenic diet may potentially explain similar autoantibody response including production of ghrelin-reactive IgG, which may contribute to the long-term regulation of host energy metabolisms by gut microbiota (57). Further studies are needed to identify potential ghrelin-mimetic antigens in gut microbiota and to analyze the effects of altered energy balance on their production.

## Author Contributions

SF wrote the manuscript, NL prepared the figures and tables, NL and RL analyzed the data and contributed to manuscript preparation.

## Conflict of Interest Statement

SF is a co-founder of TargEDys SA and NL and RL are currently its employees.
